# The effect of white matter signal abnormalities on default mode network connectivity in mild cognitive impairment

**DOI:** 10.1002/hbm.24871

**Published:** 2019-11-19

**Authors:** Zhuonan Wang, Victoria J. Williams, Kimberly A. Stephens, Chan‐Mi Kim, Lijun Bai, Ming Zhang, David H. Salat

**Affiliations:** ^1^ Athinoula A. Martinos Center for Biomedical Imaging, Department of Radiology Massachusetts General Hospital Charlestown Massachusetts; ^2^ Department of Medical Imaging The First Affiliated Hospital of Xi'an Jiaotong University Xi'an China; ^3^ Alzheimer's Clinical and Translational Research Unit, Department of Neurology Massachusetts General Hospital Charlestown Massachusetts; ^4^ The Key Laboratory of Biomedical Information Engineering, Ministry of Education, Department of Biomedical Engineering, School of Life Science and Technology Xi'an Jiaotong University Xi'an China; ^5^ Neuroimaging Research for Veterans Center VA Boston Healthcare System Boston Massachusetts

**Keywords:** cortical thickness, default mode network, mild cognitive impairment, vascular, white matter signal abnormalities

## Abstract

Regions within the default mode network (DMN) are particularly vulnerable to Alzheimer's disease pathology and mechanisms of DMN disruption in mild cognitive impairment (MCI) are still unclear. White matter lesions are presumed to be mechanistically linked to vascular dysfunction whereas cortical atrophy may be related to neurodegeneration. We examined associations between DMN seed‐based connectivity, white matter lesion load, and cortical atrophy in MCI and cognitively healthy controls. MCI showed decreased functional connectivity (FC) between the precuneus‐seed and bilateral lateral temporal cortex (LTC), medial prefrontal cortex (mPFC), posterior cingulate cortex, and inferior parietal lobe compared to those with controls. When controlling for white matter lesion volume, DMN connectivity differences between groups were diminished within bilateral LTC, although were significantly increased in the mPFC explained by significant regional associations between white matter lesion volume and DMN connectivity only in the MCI group. When controlling for cortical thickness, DMN FC was similarly decreased across both groups. These findings suggest that white matter lesions and cortical atrophy are differentially associated with alterations in FC patterns in MCI. Associations between white matter lesions and DMN connectivity in MCI further support at least a partial but important vascular contribution to age‐associated neural and cognitive impairment.

## 1 INTRODUCTION

The default mode network (DMN) is a large scale brain network comprised of disparate yet highly connected cortical regions including the medial prefrontal cortex (mPFC), inferior parietal lobe (IPL), posterior cingulate cortex (pCC), precuneus, retrosplenial cortex, medial temporal lobe (MTL), lateral temporal cortex (LTC), and anterior cingulate cortex (ACC; Buckner et al., 2009; Buckner, Andrews‐Hanna, & Schacter, 2008; Raichle et al., 2001; Yeo et al., 2011). Prior work has demonstrated striking spatial overlap between regions of the DMN and cortical areas most susceptible to Alzheimer's disease (AD)‐related pathology and neurodegeneration (Celone et al., 2006; Dickerson et al., 2004, 2005; Dickerson & Sperling, 2008), with alterations in DMN functional connectivity routinely observed among individuals with mild cognitive impairment (MCI; Ewers, Sperling, Klunk, Weiner, & Hampel, 2011; Greicius, Srivastava, Reiss, & Menon, 2004; Petrella, Sheldon, Prince, Calhoun, & Doraiswamy, 2011; Pihlajamaki, Jauhiainen, & Soininen, 2009; Sala‐Llonch et al., 2010; Sorg et al., 2007; Sperling et al., 2009; L. Wang et al., 2013), as well as those with AD (Badhwar et al., 2017; Buckner et al., 2009; Koch et al., 2015; Pievani et al., 2017; Sperling et al., 2009; C. Wang et al., 2018). Given that MCI often represents an early stage of AD (Bai et al., 2009; Deshpande & Hu, 2012; Deshpande, Santhanam, & Hu, 2011; Jiao et al., 2011) with an annual conversion rate of 10–15% (Gauthier et al., 2006; Petersen et al., 2001), a greater understanding of differential mechanisms likely contributing to early DMN network dysfunction in MCI is of interest. Because functional connectivity depends on both intact white matter pathways connecting the cortical areas, as well as the structural integrity of the cortical regions themselves, it is important to consider whether these distinct structural factors uniquely contribute to disrupted functional connectivity metrics in those with MCI.

White matter damage is common in older adults and in most cases, this represents tissue compromise of a presumed vascular origin that is often visualized on MRI as white matter signal abnormalities (WMSA; Canu et al., 2012; Carmichael et al., 2010; Maillard et al., 2012). Although the clinical manifestations of WMSA are often associated with vascular conditions such as small cerebrovascular disease (Kalheim, Bjornerud, Fladby, Vegge, & Selnes, 2017; Lindemer, Greve, Fischl, Augustinack, & Salat, 2017; Wardlaw et al., 2013; Wardlaw, Smith, & Dichgans, 2013; Wirth et al., 2017), recent work has also suggested a mechanistic role of WMSA in the development of MCI and AD (Brickman et al., 2012; Coutu et al., 2016; Lindemer et al., 2017). Notably, WMSA burden has been shown to precipitate the clinical manifestation of MCI (Luchsinger et al., 2009; Provenzano et al., 2013), as well as contribute to the conversion from MCI to AD (Lindemer et al., 2015). Increasing WMSA burden within DMN regions has been shown to contribute to frontal‐subcortical pathway disruption and associated cognitive impairment (Habes et al., 2016; Luchsinger et al., 2009; Pugh & Lipsitz, 2002), and decreased functional connectivity in MCI is further associated with reduced white matter structural connectivity (Zhou et al., 2008).

On the other hand, cortical thinning is considered a marker of neurodegenerative change in AD that is linked to amyloid/tau pathological burden independent of vascular conditions and white matter lesion burden (Coutu et al., 2017; Wirth et al., 2017; Zheng et al., 2016). Cortical atrophy patterns have been shown to distinguish MCI from normal aging, where thinning is first observed in medial temporal regions, and then spreading to association areas within medial parietal, lateral temporal, and frontal regions in tandem with AD pathological progression (Driscoll et al., 2009; McEvoy et al., 2009, 2011; Wirth et al., 2017). Importantly, these cortical atrophy patterns in MCI largely overlap with key DMN regions, with particular emphasis on temporal cortex which is shown to be the most vulnerable to cortical thinning in MCI (Driscoll et al., 2009; Fjell et al., 2009; Pfefferbaum et al., 2013; Raz, Ghisletta, Rodrigue, Kennedy, & Lindenberger, 2010). Thus, abnormal DMN connectivity among individuals with MCI may be related to cortical thinning found in regions most vulnerable to AD pathology (Dickerson et al., 2004, 2009). However, it is unclear whether the effects of white matter lesions on DMN connectivity are distinct from those of cortical atrophy among those with MCI.

The present study aimed to examine alterations in seed‐based DMN functional connectivity between individuals with neuropsychologically defined MCI and controls, and whether functional alterations were associated with two distinct measures of brain structural integrity that may be impacted by differing presumed pathological origins: white matter lesions and cortical thickness.

## 2 MATERIALS AND METHODS

### 2.1 Participants

A total of 37 older adults aged 60–80 years were enrolled in this study conducted within the Brain Aging and Dementia (BAnD) Laboratory at Massachusetts General Hospital (MGH). Participants were referred for the study through the MGH Alzheimer's Disease Research Center (MGH ADRC), enrolled from a local longitudinal cohort, or through community outreach. All participants were generally healthy and free of atypical disease processes for their age, with exclusion criteria consisting of: a history of heart disease, stroke, or kidney disease, the presence of acute or chronic major neurological conditions, uncontrolled diabetes (based on glucose levels <200 mg/dL when medicated), current use of psychoactive drugs, and contraindications to the MRI environment. All participants were fully independent in activities of daily living—and thus above clinical diagnostic thresholds for major neurocognitive disorder. This study was approved by Partners Healthcare institutional review board (IRB) and was in accordance with the Declaration of Helsinki. Informed consent was obtained from each participant.

### 2.2 Neuropsychological assessment: Classification of MCI

Enrolled participants were administered a comprehensive neuropsychological assessment battery consisting of the following measures: Mini‐Mental Status Examination (MMSE), Montreal Cognitive Assessment (MoCA), Wechsler Adult Intelligence Scale—Digit Span (WAIS‐DS), Trail Making Test—Parts A and B (TMT‐A, TMT‐B), Symbol‐Digit Modalities Test (SDMT), Hopkins Verbal Learning Test—Revised, Total Learning (HVLT‐TL) and Delayed Recall (HVLT‐DR), Brief Visuospatial Memory Test—Revised, Total Learning (BVMT‐TL) and Delayed Recall (BVMT‐DR), Weschsler Memory Scale—Logical Memory I and II (WMS‐LM I, WMS‐LM II), Delis‐Kaplan Executive Function System—Letter Fluency (DKEFS‐Letter) and Category Fluency (DKEFS‐Category), and the Stroop Color Word Interference Test. Each participant's raw neuropsychological data was first converted to standard scores based on population‐based norms published for each test, and then transformed to *z*‐scores. The MCI group was operationally defined using previously published criteria that classifies MCI by a noted impairment in two or more cognitive domains, with domain‐level impairment defined by two or more tests within that domain falling at least one *SD* below published normative values (Bondi et al., 2008, 2014; Jak et al., 2009; Stricker et al., 2013). Based on this operationalization, 20 participants were determined to be cognitively intact and formed our control group (CON), and 17 participants met criteria for MCI—all of which had noted impairment in the memory domain, suggesting an amnestic subtype of MCI.

### 2.3 MRI acquisition

All structural and functional MRI data was acquired on a 3‐Tesla Siemens Trio scanner (Erlangen, Germany) with a 12‐channel phased‐array head coil. High‐resolution T1‐weighted images were obtained for each subject with multiecho MPRAGE (van der Kouwe, Benner, Salat, & Fischl, 2008): repetition time (TR) = 2,530 ms, echo time (TE) = 1.64 ms, inversion time (TI) = 1,000 ms, flip angle = 7°, slice thickness = 1 mm, field of view (FOV) = 256 mm × 256 mm, matrix size = 256 × 256. The T2‐weighted fast spin echo sequence was acquired with: TR = 3,200 ms, TE = 425 ms, slice thickness = 1.0 mm, FOV = 256 mm × 256 mm, matrix size = 512 × 512. The resting state functional MRI (rsfMRI) scan was acquired with whole‐brain echo planar imaging (EPI) time‐series scans: TR = 3,000 ms, TE = 30 ms, flip angle = 90°, slice thickness = 3.0 mm, FOV = 216 mm × 216 mm, matrix size = 72 × 72. The scanner operator was continuously in contact with participants throughout the scanning session and participants were instructed to keep their eyes open throughout the rsfMRI acquisition immediately prior to starting the scan.

### 2.4 T1‐weighted high‐resolution structural imaging

T1‐weighted anatomical images were automatically processed to reconstruct cortical surfaces and to segment volume region‐of‐interests (ROIs) using the standard FreeSurfer processing stream (http://surfer.nmr.mgh.harvard.edu/; Dale, Fischl, & Sereno, 1999; Fischl, Sereno, Tootell, & Dale, 1999). The technical details of these procedures are described in prior publications (Fischl et al., 2002; Fischl, Salat et al., 2004; Fischl, van der Kouwe et al., 2004; Fischl, Liu, & Dale, 2001) but briefly, this process entails removal of nonbrain tissue, automated Tailarach transformation, gray/white tissue segmentation, intensity normalization, parcellation of the gray/white matter boundaries, topology correction, surface deformation, and parcellation of the cortical surface based on gyral and sulcal structures. Cortical thickness was defined as the shortest distance between vertices comprising the inner surface (gray/white boundary) and outer surface (gray/pial boundary; Fischl & Dale, 2000). All T1‐weighted images were registered onto a common surface template using a surface‐based averaging technique reliant on cortical folding patterns. All raw imaging data were inspected for artifacts and accuracy of surface boundary placement (which were manually corrected if needed) prior to analysis. Total WMSA volumes were quantified for each hemisphere using T1/T2 image data based on previously described procedure (Lindemer et al., 2015, 2017; Lindemer, Greve, Fischl, Augustinack, & Salat, 2017; Lindemer, Greve, Fischl, Salat, & Gomez‐Isla, 2018). Briefly, this procedure registers T2‐weighted images to an individual's T1‐weighted image after it has been processed through FreeSurfer's recon‐all stream. It then performs intensity normalization of modalities using a multimodal atlas and segments WMSA from normal‐appearing white matter (NAWM) using a spatial array of multimodal Gaussian classifiers as well as individual‐based heuristics (Lindemer et al., 2017). WMSAs were defined based on consensus guidelines for measurement of WM hyperintensities of presumed vascular origin (Wardlaw, Smith, Biessels, et al., 2013). Using these new WMSA labels in conjunction with all standard FreeSurfer labels, a multimodal Gaussian classifier array (MMGCA) was created that contained a matrix (T1 and T2) for each structure at each voxel in addition to spatial and neighborhood prior information. Then follow up the MMGCA procedure with several refinements designed to catch unlabeled WMSAs. These refinements rely heavily on the Mahalanobis distance (MD) of a WMSA voxel from normal‐appearing white matter (Lindemer et al., 2015).

### 2.5 Resting state fMRI processing

Resting state function MRI data were processed using FreeSurfer (Fischl, Sereno, & Dale, 1999) FS‐FAST processing stream (http://surfer.nmr.harvard.edu). Preprocessing of resting state fMRI scans utilized the FS‐FAST standard stream procedure which included motion correction, concatenation of scans across runs, masking of nonbrain tissue, registration to the anatomical image, sampling of time series fMRI data onto the surface, and surface smoothing using a full width half maximum of 15 mm.

**Figure 1:**
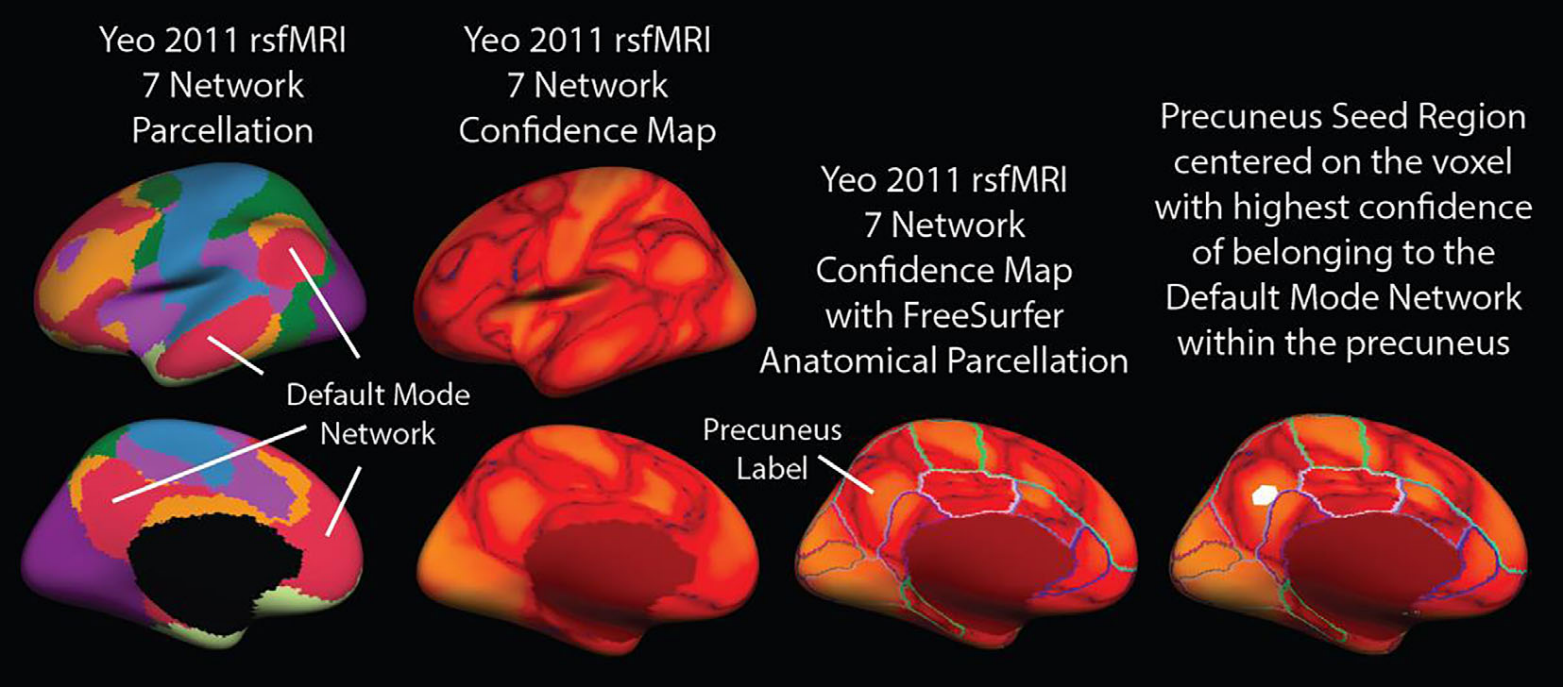
The Yeo 7‐network parcellation atlas was created using independent component analysis from over 1,000 young healthy adults and is paired with a corresponding confidence map providing an estimate for each vertex across the cortical mantle as belonging to each of seven distinct rsfMRI networks. To create each DMN seed region in standard space (using the FSaverage template), the surface‐based parcellation of the precuneus was used as a mask region to constrain the search for the vertex with the peak confidence value of belonging to the DMN within the Yeo 7Network Confidence map. Next, for each hemisphere separately, the selected precuneus vertex with the highest DMN confidence rating was dilated by a factor of 10 on the cortical surface resulting in a circular seed region, and then warped to each participant's native fMRI space to extract the seed time course for each hemisphere separately

Because our MCI group is of the amnestic subtype with a high rate of conversion to AD, we chose the precuneus as our seed region for functional connectivity analysis given that this brain area is a key anatomical hub of the DMN, and is presumed to play an important role in episodic memory, a cognitive function invariably disrupted in Alzheimer's disease (Weiler et al., 2014). As visualized in Figure 1, to perform seed‐based functional connectivity analyses, we empirically determined left and right precuneus‐seed regions based on the Yeo 7‐network parcellation atlas, which includes segmentation of the DMN (Yeo et al., 2011). The Yeo 7‐network parcellation atlas was created using independent component analysis from over 1,000 young healthy adults and is paired with a corresponding confidence map providing an estimate for each vertex across the cortical mantle as belonging to each of seven distinct rsfMRI networks. To create each DMN seed region in standard space (using the FSaverage template), the surface‐based parcellation of the precuneus was used as a mask region to constrain the search for the vertex with the peak confidence value of belonging to the DMN within the Yeo 7Network Confidence map. Next, for each hemisphere separately, the selected precuneus vertex with the highest DMN confidence rating was dilated by a factor of 10 on the cortical surface resulting in a circular seed region, and then warped to each participant's native fMRI space to extract the seed time course for each hemisphere separately.

Subject‐level voxel‐wise time series analysis was performed for each hemisphere separately to determine the correlation of the mean precuneus‐seed time course to voxel‐wise time series data sampled to each vertex along the cortical surface. In this procedure, the first four time points were discarded to account for scanner drift and data was concatenated across multiple runs and band pass filtered between 0.01 and 0.1 Hz. Correlation coefficients were calculated between the precuneus‐seed region and each vertex along the cortical mantle regressing the effects of several nuisance variables including polynomial motion correction parameters, global signal, and mean time courses obtained from segmented ventricular and white matter regions. Resultant surface‐based correlation coefficient maps reflecting precuneus‐seed connectivity across the cortex were concatenated across subjects in standard space and entered into general linear models as described below.

### 2.6 Statistical analysis

All demographic and clinical data were assessed for normality and the presence of outliers prior to statistical analysis using IBM SPSS Statistics version 24. Surface‐based neuroimaging data was analyzed using FreeSurfer's general linear modeling tool mri_glmfit. Within‐group analyses were performed using one‐sample group means (OSGM) for each group independently to demonstrate regions showing significant functional connectivity with the precuneus‐seed with and without covariates of interest (WMSA burden, cortical thickness) included in the models. Total hemispheric WMSA volume was considered as a constant regressor value across all vertex‐wise analyses, whereas cortical thickness was treated as a per‐vertex regressor such that individual cortical thickness metrics were used as a regressor at each corresponding vertex. Between‐group analyses were performed to investigate differences in DMN connectivity profiles between MCI and control groups using general linear modeling both with and without regression for WMSA volume and cortical thickness. Because the two groups were well‐matched on sex, age, and education, these demographic variables were not included as covariates for surface‐based models, but were evaluated through post hoc analyses within regions of interest showing a significant difference between groups (see Supporting Information Table S1). Vertex‐wise statistical results were thresholded at *p* < .05 and corrected for multiple comparisons using a cluster‐based procedure adapted for cortical surface analysis with a cluster‐wise alpha level of *p* < .05. The data that support the findings of this study are available from the corresponding author upon reasonable request.

## 3 RESULTS

### 3.1 Participants

**Figure 2:**
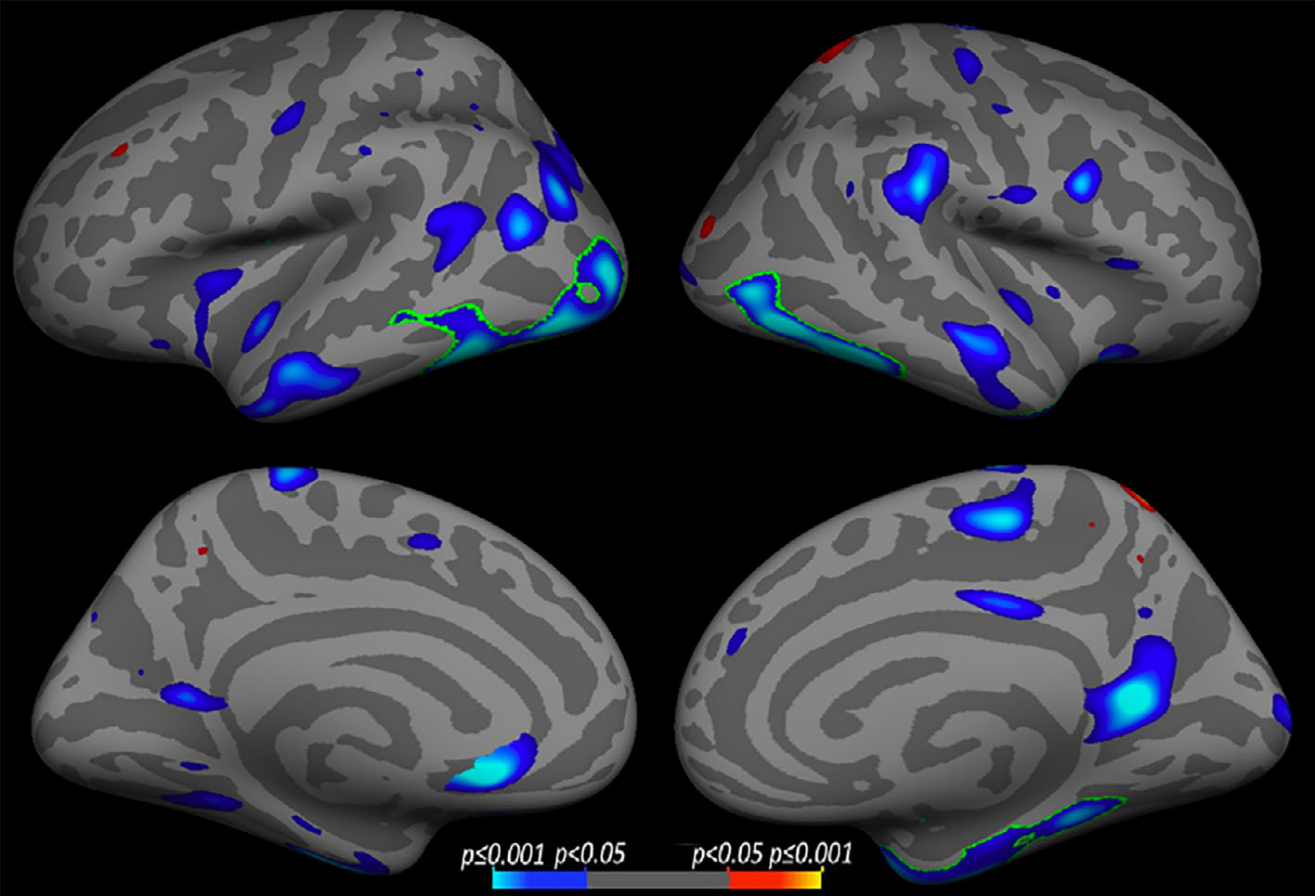
Results of whole‐brain vertex‐wise cortical thickness analysis between healthy older adult control (CON) and mild cognitive impairment (MCI) groups. Cool colors correspond to regions where the MCI had thinner cortex compared to the CON group. Warm colors correspond to the opposite contrast. Data were thresholded at *p* < .05, with a saturation of *p* < .001, green outlines indicate regions that survived after multiple comparison correction, with a cluster‐wise statistical threshold set to *p* < .05

Group characteristics are summarized in Table 1. As expected, those with MCI scored significantly lower on the MMSE (*p* < .05) and MoCA (*p* < .001) compared to controls. The two groups did not significantly differ in regard to age, years of education, or sex distribution (all *p* > .05). The group with MCI also demonstrated significantly greater total WMSA volume compared to controls (*p* < .05). Because cortical thickness was entered into models as a per‐vertex regressor, group differences in cortical thickness are demonstrated as surface models in Figure 2. Overall, individuals with MCI had significantly reduced cortical thickness relative to the CON group in bilateral lateral and medial temporal areas, visual cortex, primary somatosensory, and pCC regions, as well as the left mPFC. The result kept stable in bilateral visual cortex and right medial temporal areas after multiple comparisons.

**Table 1:** Demographic data for MCI and controls

	Cognitively healthy (*n* = 20)	MCI (*n* = 17)	*t*‐Value (*p*‐value)
Demographic			
Age (years)	68.03 ± 6.01	68.57 ± 6.15	−0.267 (.791)
Gender (M:F)	7:13	7:10	*χ* ^2^ = 0.149 (.699)
Education (years)	16.65 ± 2.70	15.29 ± 2.52	1.58 (.124)
MMSE	28.85 ± 1.46	27.59 ± 2.06	**2.17 (.037)**
MoCA	27.05 ± 2.11	23.00 ± 2.87	**4.81 (<.001)**
Neuropsychological assessment			
WAIS—DS	0.72 ± 1.07	0.61 ± 0.88	1.40 (.170)
Trails A	−0.38 ± 0.66	−1.17 ± 0.88	**3.10 (.004)**
Trails B	0.06 ± 1.00	−1.35 ± 0.86	**4.54 (<.001)**
SDMT	1.47 ± 0.96	0.23 ± 1.15	**3.66 (.001)**
HVLT‐TL	0.42 ± 0.85	−1.33 ± 0.85	**6.23 (<.001)**
HVLT‐DR	0.8 ± 1.2	−1.57 ± 1.28	**5.79 (<.001)**
BVMT‐TL	0.07 ± 0.9	−1.69 ± 0.67	**6.51 (<.001)**
BVMT‐DR	0.69 ± 0.92	−1.33 ± 0.92	**6.56 (<.001)**
WMS‐LM I	1.32 ± 0.78	0.21 ± 1.05	**3.65 (.001)**
WMS‐LM II	1.68 ± 0.85	0.52 ± 0.86	**4.06 (<.001)**
D‐KEFS letter fluency	1.28 ± 1.04	0.38 ± 1.11	**2.53 (.016)**
D‐KEFS category fluency	0.72 ± 0.61	−0.1 ± 0.65	**3.91 (<.001)**
Stroop color	0.55 ± 0.78	−0.25 ± 1.04	**2.64 (.013)**
Stroop word	0.17 ± 0.99	−0.52 ± 0.91	**2.13 (.040)**
Stroop interference	0.97 ± 0.83	−0.42 ± 0.89	**4.82 (<.001)**
WMSA volume			
LH WMSA volume (mL)	7,823 ± 10,154	17,069 ± 12,591	**−2.47 (.018)**
RH WMSA volume (mL)	7,875 ± 10,546	17,050 ± 13,166	**−2.35 (.024)**

*Note*: The bold value indicates *p* <.05 between groups.

Abbreviations: BVMT‐TL & BVMT‐DR, Brief Visuospatial Memory Test—Revised, Total Learning and Delayed Recall; DKEFS‐Letter & DKEFS‐Category, Delis‐Kaplan Executive Function System—Letter Fluency and Category Fluency, and the Stroop Color Word Interference Test; HVLT‐TL & HVLT‐DR, Hopkins Verbal Learning Test—Revised, Total Learning and Delayed Recall; LH, left hemisphere; MCI, mild cognitive impairment; MMSE, mini‐mental state examination; MoCA, Montreal Cognitive Assessment; RH, right hemisphere; SDMT, Symbol‐Digit Modalities Test; TMT‐A, TMT‐B, Trail Making Test—Parts A and B; WAIS‐DS, Wechsler Adult Intelligence Scale—Digit Span; WMS‐LM I & WMS‐LM II, Weschsler Memory Scale—Logical Memory I and II; WSMA, white matter signal abnormalities.

### 3.2 DMN connectivity

**Figure 3:**
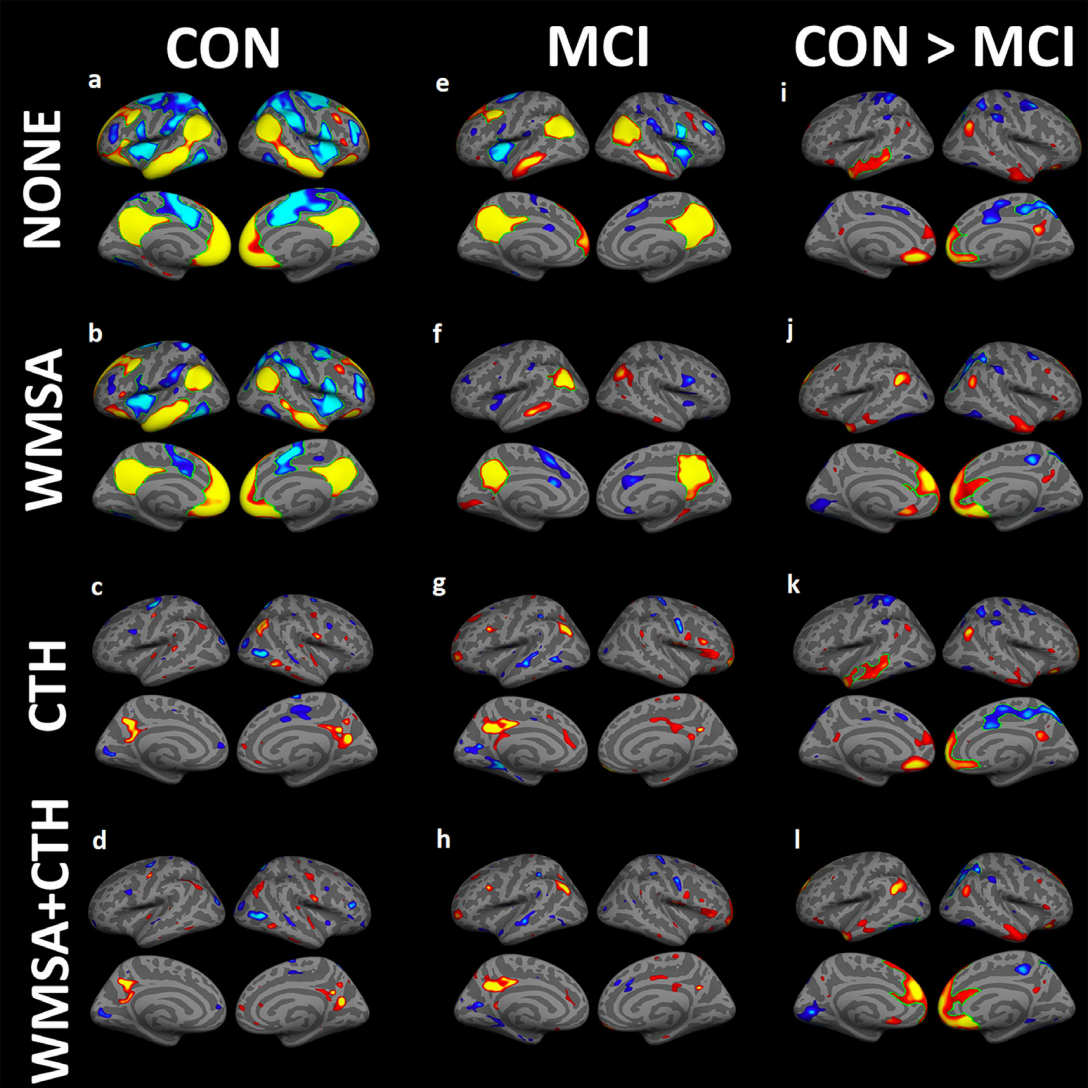
One‐sample group mean (OSGM) functional connectivity maps are shown for (1) cognitively healthy controls (CON): (a) without any regressors, (b) regressing out the effects of white matter signal abnormalities (WMSA) volume only, (c) regressing out cortical thickness (CTH) only, and (d) regressing out both WMSA and CTH. (2) Mild cognitive impairment (MCI) group: (e) without any regressors, (f) regressing out WMSA volume only, (g) regressing out cortical thickness (CTH) only, and (h) regressing out both WMSA and CTH. For the within‐group analyses (a‐h), warm colors indicate significant regional positive correlations with the precuneus‐seed, and cool colors indicate regions of anticorrelation. Between‐group analyses comparing precuneus‐seeded DMN functional connectivity maps between MCI and CON groups: (i) without any regressors, (j) regressing out WMSA only, (k) regressing out CTH only, and (l) regressing out both WMSA and CTH. Warm colors indicate regions of stronger DMN connectivity in CON compared to MCI, whereas cool colors indicate regions where the MCI group showed stronger DMN connectivity compared to the CON group. For all analyses shown, statistical thresholds were set to *p* < .05, saturation to *p* < .001, with green outlines indicating regions that survived after multiple comparison correction using a cluster‐wise statistical threshold set to *p* < .05

As shown in Figure 3, functional connectivity maps derived from an empirically‐defined precuneus‐seed showed robust positive correlations with regions classically implicated in the DMN, including bilateral mPFC, pCC, LTC, and IPL cortices across both groups. Areas of anticorrelation were also observed bilaterally in the primary somatosensory cortex, primary motor cortex, somatosensory association cortex, premotor cortex and supplementary motor cortex, inferior frontal gyrus and Broca's area. When directly comparing the functional connectivity maps between the two groups without any regressors (Figure 3i), those with MCI showed a reduced degree of functional connectivity between the precuneus‐seed and mPFC, pCC, LTC, and IPL regions compared to the CON group (*p* < .05, uncorrected). Left LTC and right mPFC and pCC clusters remained statistically significant after correcting for multiple comparisons as indicated by green outlines in Figure 3. To assess the degree to which regional differences in precuneus‐seeded functional connectivity between groups (HC vs. MCI) associated with neuropsychological performance, we performed follow‐up analyses within clusters that showed a significant difference in functional connectivity between groups that survived multiple comparison correction (left LTC, right mPFC, and pCC). The full table depicting correlation coefficients for each test is presented in Supporting Information Table S2. Overall, we observed that the degree of functional connectivity between the precuneus and the left LTC was strongly correlated with performance across multiple memory tasks: HVLT‐TL (*r* = .476, *p* < .01), HVLT‐DR (*r* = .447, *p* < .01), BVMT‐TL (*r* = .482, *p* < .01), BVMT‐DL (*r* = .483, *p* < .01), and that the degree of functional connectivity between the precuneus and right mPFC was significantly associated with tasks of executive functioning: Trails B (*r* = .409, *p* < .05) and Stroop interference (*r* = .480, *p* < .01).

When *WMSA* volume was added as a regressor in the model, there was a minimal effect on DMN connectivity maps in the cognitively healthy older adult group (Figure 3b). However, adding WMSA as a covariate significantly diminished DMN connectivity patterns in the MCI group (Figure 3f) particularly within bilateral LTC and mPFC regions, suggesting that WMSA load accounted for a notable amount of variance in connectivity strength between these regions and the precuneus node. When directly comparing DMN maps between groups while additionally controlling for WMSA burden (Figure 3j), fewer group differences were noted in bilateral LTC regions, although greater differences in functional connectivity emerged between groups in medial frontal areas such that the strength of functional connectivity was significantly decreased in the MCI group compared to controls.

When per‐vertex *cortical thickness* measures was specified as a covariate in within‐subject analyses (Figure 3c,g), both groups showed similar reductions in precuneus‐seed connectivity within IPL, pCC, LTC and mPFC regions when compared to group specific DMN maps without any regressors included (Figure 3a,e). Between‐group analyses additionally controlling for cortical thickness (Figure 3k) revealed a similar pattern of DMN connectivity differences compared to when no covariates were included (Figure 3i) and multiple comparisons result also reserved left LTC and right mPFC difference as no covariates, suggesting that variation in cortical thickness similarly impacted connectivity profiles for both groups.

Finally, after regressing out *both WMSA and cortical thickness*, between‐group differences (Figure 3l) resemble results when regressing out the effects of WMSA volume alone (Figure 3j) and bilateral mPFC did survived multiple comparisons correction, suggesting that group differences in white matter lesion burden seem to have a greater impact on functional connectivity within the DMN compared to group differences in cortical thickness (which impacted DMN connectivity in both groups similarly).

**Figure 4:**
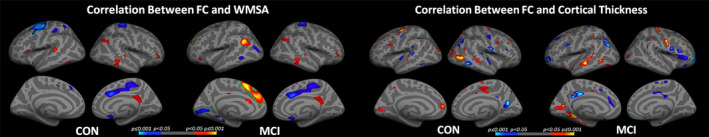
Results of within‐group correlation analyses demonstrating significant associations between precuneus‐seeded DMN functional connectivity and white matter signal abnormality (WMSA) volume and cortical thickness (CTH) for cognitively healthy controls (CON) and those with mild cognitive impairment (MCI) each group. Cool colors indicate negative correlations and warm colors indicate positive correlations, with green outlines indicating regions that survived multiple comparison correction using a cluster‐wise statistical threshold of *p* < .05

Finally, as shown in Figure 4, we additionally analyzed the correlation between DMN functional connectivity strength, WMSA volume, and cortical thickness in each group separately. In the MCI group, *WMSA volume* was positively associated with the strength of functional connectivity in left mPFC, as well as small isolated regions within the bilateral IPL and right LTC, with the left mPFC remaining significant following multiple comparisons correction. In the cognitively healthy control group, only small regions of positive correlation with WMSA was observed within the bilateral LTC, right IPL and pCC. On the other hand, correlations between DMN functional connectivity strength and *cortical thickness* revealed similar spatial patterns of effects across both MCI and control groups. Small positive correlations were found in bilateral LTC and left mPFC in controls. Meanwhile, positive correlations in the MCI group were observed in left LTC and MTL and right primary motor cortex.

## 4 DISCUSSION

The present study confirms prior findings of reduced functional connectivity in disparate regions of the DMN network in MCI relative to cognitively healthy controls (Bai et al., 2008; Chhatwal & Sperling, 2012; De Vogelaere, Santens, Achten, Boon, & Vingerhoets, 2012; Jin, Pelak, & Cordes, 2012; Li et al., 2016; Sorg et al., 2007; Weiler et al., 2014), while extending this line of work to demonstrate that regional connectivity patterns differed both within and between groups as a function of WMSA volume and cortical thickness. Both structural measures differentially influenced group differences in functional connectivity between the precuneus‐seed region and the LTC, IPL, and pCC, with WMSA exhibiting the strongest effect on DMN connectivity within mPFC regions. These findings support multiple pathologic processes contributing to network dysfunction in MCI, although the preferential effects of white matter lesion volume on medial prefrontal connectivity patterns suggests a possible vascular etiology.

To determine the relative contributions of white matter lesion burden and cortical thickness to precuneus‐seeded functional connectivity profiles, these metrics of interest were systematically added as covariates to both within‐ and between‐subject models. When WMSA volume was included as a regressor, we observed additional between‐group differences in functional connectivity metrics within the left mPFC (see Figure 3). When functional connectivity maps were derived for each group separately, the addition of WMSA as a regressor minimally altered the functional connectivity profile in the control group, although drastically decreased mPFC functional connectivity strength in those with MCI. Greater white matter lesion burden has been previously associated with impaired resting state brain connectivity within medial frontal regions in those with MCI (Zhou et al., 2015), and tract‐specific white matter damage subserving then mPFC has been related to functional connectivity disruption in patients across the continuum of AD (Taylor et al., 2017; Tullberg et al., 2004). Although WMSA are not specific to AD pathology, our prior work has shown that the regional distribution of WMSA lesions may be a critical component of AD development and progression given that regions showing a statistically significant relationship between WMSA and time‐to‐AD‐conversion are limited to the temporal and the frontal white matter (Lindemer et al., 2017). Given that the accumulation of white matter lesions are often attributed to the ischemic vulnerability of sparsely perfused vascular end zones of perforating arteries with limited collateral blood supply (Holland et al., 2008), it is likely that the observed influence of WMSA volume on DMN connectivity in MCI may be due to a presumed vascular origin (Braak & Braak, 1991; Lindemer, Greve, Fischl, Augustinack, & Salat, 2017; Wardlaw, Smith, Biessels, et al., 2013; Wardlaw, Smith, & Dichgans, 2013). White matter lesion distribution tends to spatially overlap with hubs of the DMN, presumably contributing to frontal‐subcortical pathway disruption and associated cognitive impairment (Habes et al., 2016; Luchsinger et al., 2009; Pugh & Lipsitz, 2002). Frontal brain areas are particularly vulnerability to vascular insult and the accumulation of WMSA burden in frontal regions is more closely tied to cognitive functioning compared to local patterns of cortical atrophy (Tullberg et al., 2004).

Although WMSA burden reduced mPFC DMN functional connectivity preferentially in the MCI group, the effects of cortical thickness on connectivity profiles appeared similar across the two groups. After controlling for the effects of cortical thickness on DMN connectivity, both controls and those with MCI demonstrated a dramatic reduction in precuneus‐seed‐based connectivity across all regions of the DMN. Thus, functional connectivity within the DMN is largely dependent on the structural integrity of the cortex, regardless of cognitive status. This notion is well supported by studies demonstrating both structural and functional aspects of the DMN are affected in healthy aging without demonstrated evidence of accompanying cognitive impairment (Addis, Roberts, & Schacter, 2011; Andrews‐Hanna et al., 2007) as well as in patients with MCI/AD (Fjell et al., 2014; Greicius et al., 2004; Jones et al., 2011; Walhovd et al., 2010). One possible explanation for this similar decline in functional connectivity across both groups after regressing cortical thickness is that regions within the DMN tend to consist of higher‐level association areas that are most vulnerable to age‐related decline and atrophy (Fjell et al., 2014; Lustig et al., 2003), such that age‐related cortical thinning likely exerts a more global impact on DMN functional connectivity compared to the regional specificity of WMSA accumulation.

Overall, we observed a significant reduction in DMN functional connectivity with precuneus‐seed regions after regressing out the effects of cortical thickness across both healthy aging and those with MCI. However, only those with MCI demonstrated a significant reduction in DMN connectivity when white matter lesion volume was included as a regressor. Taken together, it is likely that the effect of cortical atrophy on DMN functional connectivity may occur in both normal aging as well as in MCI populations. However, the specificity of white matter damage was more likely to affect the functional connectivity of MCI individuals, particularly in mPFC regions. Prior work has shown evidence of both increased and decreased DMN connectivity in MCI populations compared to healthy controls. The present results may help reconcile such discrepancies by highlighting the extent to which underlying white matter pathology may attenuate the strength of functional connectivity in MCI, particularly in medial frontal regions. We plan to further explore this line of work in a larger population, as well as across a greater spectrum of cognitive impairment including overt AD.

Our study is limited by its cross‐sectional design, which precludes inference that the functional connectivity changes we found would necessarily predict progression toward AD. Furthermore, MCI is a heterogeneous condition that likely reflects a range of underlying pathology aside from AD and it is possible that some subjects in our cohort had cognitive impairments due to other etiologies. Given the limited sample size of the present study, replicating the observed effects in larger cohorts is of interest, and future work in this area should investigate whether longitudinal progression of WMSA load or cortical atrophy can successfully predict declines in DMN connectivity. Longitudinal studies would also provide insight into the temporal order of gray versus white matter degenerative effects on DMN connectivity profiles. Finally, the definition of white matter lesions is still inconsistent in the literature and a standardized approach to quantitative or semiquantitative white matter lesion segmentations are lacking.

We demonstrate reduced DMN functional connectivity in those with MCI compared to cognitively healthy older adults, the extent to which was differentially regionally related to white matter lesion volume and cortical atrophy. White matter damage may play a crucial role in DMN disruption and cognitive impairment in MCI, as supported by findings of preferential associations between altered mPFC connectivity and WMSA burden in those with MCI but not controls. On the other hand, variation in cortical thickness similarly impacted DMN connectivity across both groups. These findings suggest that multiple pathologic processes contribute to network dysfunction in MCI and associations with white matter lesions may indicate a vascular etiology to subtle impairment in MCI.

## CONFLICT OF INTEREST

The authors declared no potential conflicts of interest with respect to the research, authorship, and/or publication of this article.

## AUTHOR CONTRIBUTIONS

Z.W., D.H.S.: study concept and design. K.A.S.: acquisition of the data. Z.W., V.J.W., K.A.S., C.M.K.: analysis of the data. Z.W., V.J.W., L.B., D.H.S.: interpretation of the data. Z.W.: drafting of manuscript. Z.W., V.J.W., L.B., M.Z., D.H.S.: revision of the manuscript.

## Supporting information


**Table S1** ROI follow‐up regression models controlling for demographic variables.

**Table S2** Correlation between ROI‐extracted functional connectivity and neuropsychological performance.

**Figure S1** Differences in functional connectivity between HC>MCI with and without global signal regression.

## Data Availability

The data that support the findings of this study are available from the corresponding author upon reasonable request.
