# Supplementary material for: The effect of white matter signal abnormalities on default mode network connectivity in mild cognitive impairment
Source: Hum Brain Mapp. 2019 Nov 19;41(5):1237–48. doi: 10.1002/hbm.24871 (PMC7267894; doi:10.1002/hbm.24871)
Supplement: Supplementary file 1 — Table S1 ROI follow‐up regression models controlling for demographic variables. [file HBM-41-1237-s001.docx]

Table S1. ROI follow-up regression models controlling for demographic variables.

| **ROI** | **Group** | **Sex** | **Group x Sex** | **Age** | **Education** |
| --- | --- | --- | --- | --- | --- |
| LH LatTemp | F(1,31)=9.337, | F(1,31)=1.095, | F(1,31)=0.558, | F(1,31)=0.628, | F(1,31)=0.890, |
|  | p=0.005 | p=0.303 | p=0.461 | p=0.434 | p=0.353 |
| RH vmPFC | F(1,31)=12.408, | F(1,31)=0.045, | F(1,31)=2.439, | F(1,31)=0.056 | F(1,31)=0.447, |
|  | p=0.001 | p=0.834 | p=0.128 | p=0.814 | p=0.509 |
| RH pCC | F(1,31)=10.317, | F(1,31)=0.305, | F(1,31)=0.365, | F(1,31)=0.116, | F(1,31)=0.208, |
|  | p=0.003 | p=0.585 | p=0.550 | p=0.736 | p=0.652 |
| RH=Right Hemisphere; LH=Left Hemisphere; LatTemp=Lateral Temporal; vmPFC=ventromedial prefrontal cortex; pCC=posterior cingulate cortex | | | | | |
