# Supplementary material for: The effect of white matter signal abnormalities on default mode network connectivity in mild cognitive impairment
Source: Hum Brain Mapp. 2019 Nov 19;41(5):1237–48. doi: 10.1002/hbm.24871 (PMC7267894; doi:10.1002/hbm.24871)
Supplement: Supplementary file 2 — Table S2 Correlation between ROI‐extracted functional connectivity and neuropsychological performance. [file HBM-41-1237-s002.docx]

Table S2. Correlation between ROI-extracted functional connectivity and neuropsychological performance.

|  | Mean ROI Functional Connectivity with Precuneus Seed | | |
| --- | --- | --- | --- |
|  | LH LTC | RH mPFC | RH pCC |
| CDR | -0.486^**^ | -0.486^**^ | 0.313 |
| MMSE | 0.516^**^ | 0.295 | -0.306 |
| MoCA | 0.467^**^ | 0.381^*^ | -0.396^*^ |
| WAIS –DS | 0.441^**^ | 0.318 | -0.213 |
| Trails A | 0.398^*^ | 0.229 | -0.295 |
| Trails B | 0.267 | 0.409^*^ | -0.425^**^ |
| SDMT | 0.117 | 0.278 | -0.333^*^ |
| HVLT-TL | 0.476^**^ | 0.347^*^ | -0.401^*^ |
| HVLT-DR | 0.447^**^ | 0.273 | -0.282 |
| BVMT-TL | 0.482^**^ | 0.475^**^ | -0.468^**^ |
| BVMT-DR | 0.483^**^ | 0.417^*^ | -0.377^*^ |
| WMS LM I | 0.239 | 0.293 | -0.332^*^ |
| WMS LM II | 0.253 | 0.194 | -0.349^*^ |
| D-KEFS Letter Fluency | 0.277 | 0.318 | -0.355^*^ |
| D-KEFS Category Fluency | 0.352^*^ | 0.270 | -0.303 |
| Stroop Color | 0.205 | 0.255 | -0.339^*^ |
| Stroop Word | 0.426^**^ | 0.235 | -0.284 |
| Stroop Interference | 0.290 | 0.480^**^ | -0.392^*^ |
| MoCA: Montreal Cognitive Assessment, MMSE: Mini-Mental State Examination, WAIS-DS: Wechsler Adult Intelligence Scale – Digit Span, TMT-A, TMT-B: Trail Making Test – Parts A and B, SDMT: Symbol-Digit Modalities Test, HVLT-TL & HVLT-DR: Hopkins Verbal Learning Test – Revised, Total Learning and Delayed Recall, BVMT-TL & BVMT-DR: Brief Visuospatial Memory Test – Revised, Total Learning and Delayed Recall, WMS-LM I & WMS-LM II: Weschsler Memory Scale – Logical Memory I and II, DKEFS-Letter & DKEFS-Category: Delis-Kaplan Executive Function System – Letter Fluency and Category Fluency, and the Stroop Color Word Interference Test. LH: Left Hemisphere, RH: Right Hemisphere, WSMA: white matter signal abnormalities; *p<0.05; **p<0.01 | | | |
