# Supplementary figures and images for: The effect of white matter signal abnormalities on default mode network connectivity in mild cognitive impairment
Source: Hum Brain Mapp. 2019 Nov 19;41(5):1237–48. doi: 10.1002/hbm.24871 (PMC7267894; doi:10.1002/hbm.24871)

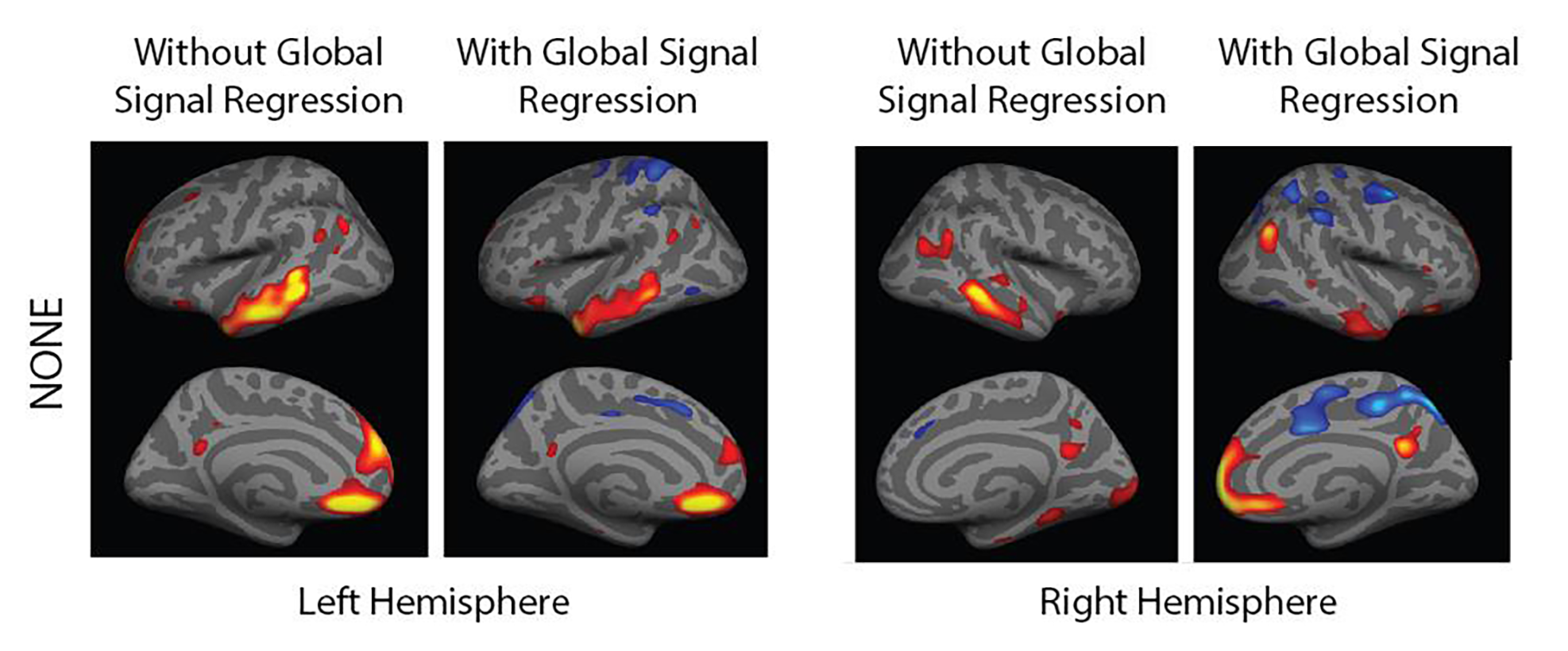

Supplement: Supplementary file 3 — Figure S1 Differences in functional connectivity between HC>MCI with and without global signal regression. [file HBM-41-1237-s003.tif]
